# Enhanced home palliative care could reduce emergency department visits due to non-organic dyspnea among cancer patients: a retrospective cohort study

**DOI:** 10.1186/s12904-021-00713-6

**Published:** 2021-03-13

**Authors:** Hua-Shui Hsu, Tai-Hsien Wu, Chin-Yu Lin, Ching-Chun Lin, Tsung-Po Chen, Wen-Yuan Lin

**Affiliations:** 1 Department of Family Medicine and Social Medicine, School of Medicine, College of Medicine, China Medical University, Taichung, Taiwan; 2 Department of Family Medicine, China Medical University Hospital, Taichung, Taiwan; 3 Department of Palliative Medicine, China Medicinal University Hospital, Taichung, Taiwan; 4 Department of Medical Research, China Medical University Hospital, Taichung, Taiwan; 5 Department of Nursing, China Medical University Hospital, Taichung, Taiwan

**Keywords:** Dyspnea, Home palliative care, Emergency department

## Abstract

**Background:**

Dyspnea is a common trigger of emergency department visits among terminally ill and cancer patients. Frequent emergency department (ED) visits at the end of life are an indicator of poor-quality care. We examined emergency department visit rates due to dyspnea symptoms among palliative patients under enhanced home palliative care.

**Methods:**

Our home palliative care team is responsible for patient management by palliative care specialists, residents, home care nurses, social workers, and chaplains. We enhanced home palliative care visits from 5 days a week to 7 days a week, corresponding to one to two extra visits per week based on patient needs, to develop team-based medical services and formulate standard operating procedures for dyspnea care.

**Results:**

Our team cared for a total of 762 patients who exhibited 512 ED visits, 178 of which were due to dyspnea (mean ± SD age, 70.4 ± 13.0 years; 49.4% male). Dyspnea (27.8%) was the most common reason recorded for ED visits, followed by pain (19.0%), GI symptoms (15.7%), and fever (15.3%). The analysis of Group A versus Group B revealed that the proportion of nonfamily workers (42.9% vs. 19.4%) and family members (57.1% vs. 80.6%) acting as caregivers differed significantly (*P* < 0.05). Compared to the ED visits of the Group A, the risk was decreased by 30.7% in the Group B (*P* < 0.05).

**Conclusions:**

This study proves that enhanced home palliative care with two additional days per week and formulated standard operating procedures for dyspnea could significantly reduce the rate of ED visits due to non-organic dyspnea during the last 6 months of life.

**Supplementary Information:**

The online version contains supplementary material available at 10.1186/s12904-021-00713-6.

## Background

Home is the most common place of death for advanced cancer patients and an important site of palliative care in Taiwan and elsewhere [1]. The aim of palliative home care service is to improve the quality of life of patients with cancer and families without raising the total costs of care [2, 3]. A palliative care team, including specialist doctors, nurses, social workers, nutritionists and chaplains, gives caregivers indispensable medical and emotional assistance so that they can smooth the transition of palliative care from the hospital to home [4]. Otherwise, patients often seek urgent medical services for the emergency department (ED) [5].

Frequent emergency department visits at the end of life are an indicator of poor-quality care [6]. About four-fifths of palliative care patients with advanced cancer have repeated visits to the emergency department during the final 6 months of life [7]. However, nearly one-fourth of ED visits by palliative care patients may be avoidable [8]. For family members, because their clinical judgment abilities are not sufficient, patients tend to have more than one ED visit for the same condition [9].

Dyspnea is one of the most common refractory symptoms in patients with advanced diseases, including cancer and noncancer populations [10]. Previous studies show that the prevalence of dyspnea is greater than 50% among terminally ill patients and the cancer population [11, 12]. Dyspnea intensity and prevalence enhanced with disease progression, especially at the end of life, and it may leave devastating impacts on family and caregivers [7, 13, 14].

Dyspnea, which is a complex symptom resulting from organic causes and psychologic distress, calls for comprehensive evaluation and management of the condition [15]. Anxiety and panic are strongly correlated with psychological dyspnea [16]. Oxygen therapy and low-dose sustained-related opioids play some roles in refractory dyspnea, and nonpharmacologic treatment can provide some benefits [17, 18]. A specialist palliative care team is necessary for managing patients with incurable dyspnea. In Taiwan, several medical centers have established well-trained home palliative care teams to provide medical, psychosocial, and spiritual support for terminally ill patients and their families. Early integration of palliative care could improve quality of life by supporting patients through complex physical, psychosocial, and spiritual issues [19–21].

However, few studies have focused on strategies for decreasing suffering from dyspnea through home palliative care. The aim of the present study was to compare two different approaches for dyspnea relief provided by our home palliative care team. These findings may reveal how the burdens of dyspnea patients can be reduced by home palliative care at the end of life and help promote successful experiences in future clinical practice.

## Methods

### Study design

This study was a retrospective cohort study conducted at China Medical University Hospital, Taichung, Taiwan. Our patients who died with a primary diagnosis of cancer were from the linked hospital administrative databases during 2016–17.

### Study setting

Cancer patients were referred from outpatient departments, oncology wards, and palliative care wards in China Medical University Hospital for consecutive home palliative care. On referral to home palliative care agency, the case manager recorded basic information of the patient, such as sex, age, medical history, pathological condition, medication records, catheter location, emotion, family support and medical use location. The specialist home palliative care nurses then visited the home to evaluate home care needs, care plan, and visit frequency, caring for patient until death.

Our team members include family physicians, home care nurses, social workers, and chaplains. Our team-based services provided: a 24-h call line is available for phone consultations in the home care team’s office; according to the patients’ and family caregivers’ requirements and clinical conditions, our home care nurse arranged a visit once or twice a week, and family physician would be arranged a visit with nurse once a month. During home visits, our home care nurse would be performed consciousness assessment, vital signs measurement (eg, body temperature, pulse rate, respiration rate, blood pressure and oxygen saturation), pain assessment (eg, visual analog scale and critical care pain observation tool) and adjustment of drug (eg, tramadol, morphine and fentanyl patch), respiratory tract symptom assessment (eg, wheeze, gasp, cough, chest tightness and chest pain), gastrointestinal symptom assessment (eg, abdominal pain, nausea, vomiting, diarrhea and bowel movement frequency), urinary tract symptoms assessment (eg, urine color, frequency and volume), nutritional status and assessed the intravenous fluid supplement, catheter renewal (eg, endotracheal tube, nasogastric tube and urinary catheter), wound or ostomy nursing, as well as blood test. In addition, we offered formal education information sheets on dyspnea management to family caregivers to help them obtain organized content for home care (Table 1).

**Table 1:** Patient instructions for family caregiver

Phase 1. For restless and anxious individuals with respiration rate less than 20 times per min, follow the steps below:	
1. Check if he or she is feeling unwell.	
2. Use a fan or open the window to improve air circulation [22].	
3. Massage the acupuncture points Lu 10 (Yuji), Lu 7 (Lieque) and ST36 (Zu San Li) or have him or her try to meditate [23, 24].	
4. Refrain from straining when urinating or defecating.	
Phase 2. For individuals with respiration rate between 20 and 24 times per min, follow the steps below:	
1. Check vital signs.	
2. Check if he or she has been taking medications as prescribed.	
3. In case of thick sputum, use an ultrasonic nebulizer for steam inhalation.	
4. If necessary, clear sputum with sputum suction therapy.	
5. Breathe air in from the nose and breathe out from the mouth with a pursed lip.	
6. Prepare to rent a medical oxygen generator in case oxygen therapy is needed.	
7. Stay with the patient.	
8. Sit in a reclined position or lie down with head elevated with cushioning at a 30–45 degrees angle.	
9. Take extra precaution to protect the oral mucosa for mouth breathers.	
Phase 3. For individuals with respiration rate more than 24–28 times per min or with sighing respiration (deep breaths), follow the steps below:	
1. Check vital signs.	
2. Check consciousness.	
3. Check for death rattles.	
4. Provide oxygen therapy.	
5. Consider giving medication for management of shortness of breath.	
6. Call the palliative home-care nurse.	

Please check the box after each step is completed, so the palliative home-care nurse will have a better understanding of the patient’s conditions

### Selection criteria

There are two different home palliative care services in this study. During 2016, we performed basic home palliative care service (Group A) for all patients. During 2017, we performed enhanced home palliative care service (Group B) for all patients.

#### Group a

The basic home palliative care visits occurred 5 days a week (Monday to Friday).

#### Group B

In addition to basic home palliative care, the enhanced home palliative care extended the service time (seven days a week), and trained our home care nurse with formulated standard operating procedures for dyspnea care (Table 2).

**Table 2:** Standard operating procedures for dyspnea and excessive sputum with difficult expectoration in Group B patients

Learning Goals	
1. Being able to list out current medications.	
2. Assessment of dyspnea, labored breathing, excessive sputum with difficult expectoration, and symptoms of deoxygenation.	
3. Assessment of sputum amount, color, and odor.	
4. Management of acute dyspnea.	
5. Management of excessive sputum and difficult expectoration.	

Note. A. Lung primary cancer or metastases, compression of tumor mass on the airway, pulmonary infection, failure to clear sputum, heart failure, anemia and other situations may lead to dyspnea

B. Timely management involves minimizing patient’s fear, easing tension, and using simple yet appropriate methods to regain airway patency

### Data source

This study used ED clinical data of cancer patients who received home palliative care during the final 6 months of life. The reasons for ED visits were anemia, altered mental status, catheter-related events, dyspnea, falls, fever, N/V or other GI symptoms, pain, and tumor bleeding/complications. Dyspnea is a common symptom among the ill and older patients during the last 3 days of life [25]. Patients (> 18 years old) with dyspnea who visited the ED within 3 days prior to death were excluded from this study. The following data were extracted for each eligible participant: sex, age, educational status, religion, place of care, type of caregiver, number of nurse home visits and calls, major reasons for ED visit, and site of death.

### Statistical analysis

A general linear model was used to compare group differences. All statistical analyses were performed using SPSS version 21.0 (SPSS, Inc., IBM Company, Chicago, IL, USA). All *P*-values were based on two-tailed tests with statistical significance set at < 0.05.

## Results

**Fig. 1:**
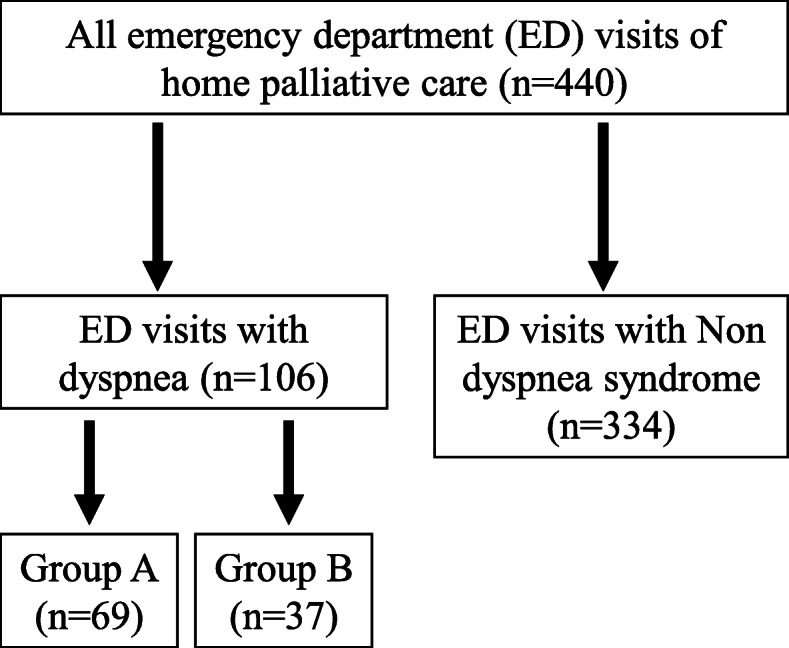
Flowchart of study subjects

During the study period from January 1, 2016 to December 31, 2017, our home palliative care team served a total of 762 patients with cancer: 374 were in Group A, and 388 were in Group B. A total of 440 ED visits were recorded, among which 106 were attributed to dyspnea (Fig. 1). Of the 89 patients (106 ED visits), 20 (22%) were classified as lung cancer in situ and 21(24%) were lung metastasis (Supplementary Table 1).

### Demographics of ED patients with dyspnea

Table 3 depicts the characteristics of ED visits attributed to dyspnea under our home palliative care services. The total number of ED visits due to dyspnea was 106 (69 from the Group A and 37 from the Group B). The mean ± SD age of subjects was 70.4 ± 13.0 years, 49.4% male. The majority of caregivers of patients were migrant workers, companions, offspring and siblings of the patient. Analysis of home palliative care revealed that the proportion of nonfamily workers (42.9% vs. 19.4%) and family members (57.1% vs. 80.6%) acting as caregivers was significantly different between Group A and B (*P* < 0.05). In multivariable logistic regression analysis adjusted for each covariate, the types of caregivers was an independent factor (OR = 3.29. 95%CI = 1.01–10.78, *P* < 0.05) in enhanced home palliative care service (Supplementary Table 2). There was no significant difference in the proportion of patient deaths at home in Group A and Group B (24.5% vs 33.3%, *P* = 0.39). In unadjusted and adjusted logistic regression analysis, there were also no significant associations between home palliative care service group and site of death (Supplementary Table 2).

**Table 3:** Characteristics of ED patients with dyspnea attributed to the home palliative care patients with cancer

Characteristic	All sample	Group A	Group B	*P*-value^*^
Patients, No.	89	57	32	
ED visits	106	69	37	
Man, No (%)	44 (49.4)	27 (47.4)	17 (53.1)	0.60
Age, mean ± SD, yr	70.4 ± 13.0	69.3 ± 13.1	72.6 ± 12.7	0.31
Grade of school completed, yr	8 ± 4.8	8.4 ± 4.4	7.3 ± 5.5	0.32
Home care services				
Telephone nurse per weeks	1.3 ± 1.2	1.4 ± 0.8	1.4 ± 2.0	0.95
Visiting nurse per weeks	0.77 ± 0.89	0.75 ± 0.4	0.98 ± 1.8	0.32
Types of Caregivers				
Family	57 (65.5)	32 (57.1)	25 (80.6)	**0.027**
Nonfamily	30 (34.5)	24 (42.9)	6 (19.4)	
Site of death				
Home	23 (27.7)	13 (24.5)	10 (33.3)	0.39
Hospital	60 (72.3)	40 (75.5)	20 (66.7)	

* General linear model for continuous variables, Chi-square test for categorical variables for the different palliative home care methods

### Association between improved care quality and ED visits

**Fig. 2:**
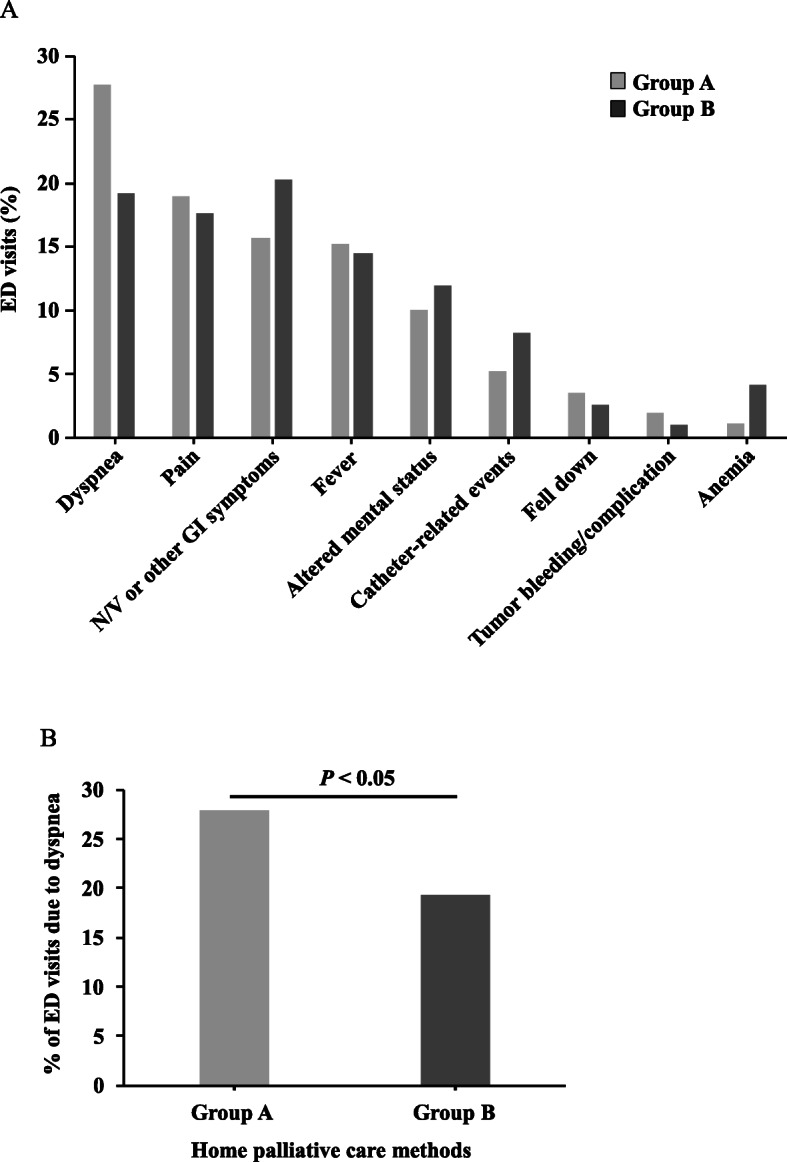
Reasons for visits to the emergency department (ED) by home palliative care patients with cancer. **a** all reasons of ED visits **b** dyspnea of ED visits

Figure 2a summarizes the numbers and major reasons for ED visits. The total ED visits in Group A and Group B were 248 and 192, respectively. In Group A, dyspnea (27.8%) was the most common reason recorded for ED visits, followed by pain (19.0%), GI symptoms (15.7%), and fever (15.3%). Through improved home palliative care intervention, the percentage of ED visits for dyspnea was reduced in Group B compared the Group A. As shown in Fig. 2b, the risk was significantly reduced by 30.7% in Group B (*P* < 0.05).

## Discussion

During the last 6 months of life in cancer patients receiving palliative home care, when additional home palliative care team interventions on the weekend were considered, ED visits were significantly reduced by 30.7% (*P* < 0.05) compared to standard palliative care. This study indicates that effective home palliative care reduced ED visits related to controllable dyspnea.

In addition to the differences in the pathophysiology of various diseases, dyspnea is strongly correlated with mood distresses, such as anxiety, panic, and fear of suffocation, which may increase respiratory effort. In 2009, the American Thoracic Society indicated that palliative management of dyspnea crisis most commonly occurs in unprepared and overwhelmed family-caregivers and in chaotic settings [26]. Because dyspnea should be assessed by the individual experiencing it, the medical care team must understand the expectations and apprehensions of the patients and caregivers [27–29]. Given that dyspnea is multifactorial, a beneficial treatment strategy should include interventions to reduce anxiety and other types of psychological distress [30]. In this study, detailed education sheets without bedside evaluation by professional medical staff could cause anxiety and confusion among caregivers, resulting in increased ED visits.

Some longitudinal surveys observed that over half of all geriatric patients with advanced illness visit the ED during their last month of life and that repeat visits are common [31, 32]. In the enhanced palliative home care of this study, we extended working hours to Saturday and Sunday (8 am to 6 pm) for palliative home care. After we applied more frequent, comprehensive home palliative visits, ED visits due to controllable dyspnea episodes were significantly reduced by 30.7% (*P* < 0.05) compared to standard working hours; overall ED visits were also significantly decreased. Multiple studies have shown similar results, i.e., that patients receiving end-of-life nursing compared to standard nursing exhibited decreased ED visits during the last 6 months of life [33, 34].

ED visits can be a source of misery because of the crowded environment in hospitals, high patient–nurse ratios, and long wait times, all of which can exhaust patients and their families. When death is imminent, ED is not the preferred place for the death of patients [35]. Delgado–Guay and colleagues found that nearly one-fourth of all ED visits by patients with advanced cancer receiving palliative care could be avoided [8].

Moreover, increased intensity of home palliative care visits provided not only pharmacological consultation but also nonpharmacological interventions for dyspnea, such as improved air flow using a fan, use of religious breathing exercises, adoption of a calm, reassuring attitude toward patients, comfortable positioning, a humane environment, and sustained safety to patients and caregivers. Similar to the findings of Sarmento VP and colleagues, coaching caregivers and enhancing the experiences of patients and families are key factors in improving home hospice care [22].

In Japan [23] and US [24], the preferred place to die is always home, and accommodating this preference is the goal in end-of-life care [31]. In this study, success was reflected not only by the achievement of the reduced ED visits but also by the significant increase in proportion of families who participated in care responsibilities instead of nonfamily caregivers (Table 1, *P* < 0.05).

### Limitations

This study has several limitations. First, our hospital, a medical center, is located in an urban setting, and the patients we served lived within the area. Therefore, the present findings do not reflect the accessibility of home palliative visits in rural areas. Second, this study was conducted in only one hospital, and replicating the outcomes in other institutions is important. Finally, further analysis was not conducted on the causes of dyspnea or their relationship with the patients’ cancers because our study sample size was small. In future work, detailed evaluations with prolonged observation are recommended.

## Conclusions

This study reveals that enhanced intensity of home palliative care visits significantly reduces the number of ED visits due to non-organic dyspnea at the end of life. Dyspnea is multifactorial, and an integrated treatment strategy should include interventions to reduce anxiety and other psychological distresses.

## Supplementary Information

**Additional file 1: Supplementary Table 1**. Cancer types of home palliative care patients with dyspnea. **Supplementary Table 2**. Logistic regression models of home palliative care services in reducing emergency department visits based on each covariate.

## Data Availability

Due to privacy and ethical concerns, neither the data nor the source of the data can be made available.

## Abbreviation

ED: Emergency Department
