# Supplementary material for: Enhanced home palliative care could reduce emergency department visits due to non-organic dyspnea among cancer patients: a retrospective cohort study
Source: BMC Palliat Care. 2021 Mar 13;20:42. doi: 10.1186/s12904-021-00713-6 (PMC7956106; doi:10.1186/s12904-021-00713-6)
Supplement: Supplementary file 1 — Additional file 1: Supplementary Table 1. Cancer types of home palliative care patients with dyspnea. Supplementary Table 2. Logistic regression models of home palliative care services in reducing emergency department visits based on each covariate. [file 12904_2021_713_MOESM1_ESM.docx]

Supplementary Table 1. Cancer types of home palliative care patients with dyspnea.

| Cancer | Total | Group A | Group B |
| --- | --- | --- | --- |
| Lung cancer, No (%) |  |  |  |
| In situ | 20 (22) | 14 (25) | 6 (19) |
| Metastasis | 21 (24) | 13 (23) | 8 (25) |
| Other cancers | 48 (54) | 30 (53) | 18 (56) |

Supplementary Table 2. Logistic regression models of home palliative care services in reducing emergency department visits based on each covariate.

|  | Univariate analysis | | |  | Multivariate analysis | | |
| --- | --- | --- | --- | --- | --- | --- | --- |
| Covariates | OR | 95%CI | *P* value |  | OR | 95%CI | *P* value |
| Gender, women vs man | 0.79 | 1.33-1.89 | 0.60 |  | 1.32 | 0.43-3.96 | 0.63 |
| Age, year | 1.02 | 0.98-1.05 | 0.31 |  | 1.04 | 0.99-1.09 | 0.17 |
| Grande of school completed, years | 0.95 | 0.87-1.05 | 0.32 |  | 1.00 | 0.87-1.14 | 0.94 |
| Telephone nurse per weeks | 1.04 | 0.76-1.41 | 0.82 |  | 0.38 | 0.11-1.32 | 0.13 |
| Home visiting nurse per weeks | 1.21 | 0.79-1.86 | 0.39 |  | 3.60 | 0.78-16.57 | 0.10 |
| Types of caregivers, family vs nonfamily | 2.68 | 0.99-7.22 | 0.051 |  | 3.29 | 1.01-10.78 | **0.049** |
| Site of death, home vs hospital | 1.54 | 0.58-4.12 | 0.39 |  | 0.97 | 0.31-3.08 | 0.96 |
